# Multifocality and opportunity structure: towards a mixed embeddedness model for transnational migrant entrepreneurship

**DOI:** 10.1186/s40878-021-00270-0

**Published:** 2022-01-10

**Authors:** Giacomo Solano, Veronique Schutjens, Jan Rath

**Affiliations:** 1 Migration Policy Group, 1030 Brussels, Belgium; 2 Department of Sociology and Social Research, University of Milan-Bicocca, 20126 Milan, Italy; 3 Amsterdam Institute for Social Science Research, University of Amsterdam, 1018 WV Amsterdam, The Netherlands; 4 Faculty of Geosciences, Utrecht University, 3584 CB Utrecht, The Netherlands

**Keywords:** Migrant entrepreneurship, Mixed embeddedness, Entrepreneurial opportunities, Transnational entrepreneurship, Transnationalism, Multifocality

## Abstract

This article addresses transnational migrant entrepreneurship, which refers to migrants involved in cross-border entrepreneurial activities. Previous models and concepts in migrant entrepreneurship studies have not fully succeeded in recognising the role played by differential groups and places in the pursuit of opportunities by transnational migrant entrepreneurs. This is due to a tendency to focus on the country of residence as well as on the inclination to view migrant entrepreneurs as members of a coherent ethnic or national group. To help fill this gap, we propose a new model combining the concept of multifocality, covering the simultaneous involvement of migrant entrepreneurs in both multiple places *and* multiple groups, with group modes of behaviour as an additional dimension influencing the opportunity structure. The case of Moroccan transnational entrepreneurs in Amsterdam shows that the role of multifocality in place, in combination with group modes of behaviour, is critical when it comes to pursuing entrepreneurial opportunities.

## Introduction

This article addresses transnational migrant entrepreneurship, which refers to the involvement of migrants in entrepreneurial activities across national borders (Drori et al., 2009), and looks at how migrant entrepreneurs pursue opportunities to develop transnational businesses. Thanks to the advancement of communication technologies—notably e-mail, Skype, Facebook, Twitter, and other social media etc.—and the widespread availability of relatively cheap travel on a large scale, people can access information about different places and contexts, and even create and actively maintain social relations all over the world (Levitt & Jaworsky, 2007; Vacca et al., 2018; Vertovec, 2004). This obviously also holds true for migrant entrepreneurs and their firms. Migrant entrepreneurs have the opportunity to develop entrepreneurial activities in their country of residence, but with multifaceted links with people and businesses abroad: so-called transnational migrant entrepreneurship. Although statistical data are scarce, past studies have shown that a significant, and increasing, number of migrant entrepreneurs have developed transnational business activities (Brzozowski et al., 2017; Elo & Minto-Coy, 2019; Harima & Baron, 2020; Liu et al., 2019; Portes & Yiu, 2013; Portes et al., 2002; Rusinovic, 2008; Sandoz et al., 2021; Solano, 2016a).

For transnational entrepreneurs, the pursuit of opportunities across borders to develop their business is key (Smans et al., 2014). As Shane and Venkataraman (2000) stated, creating, identifying and seizing business opportunities is the basis of every business. According to these authors, the study of every entrepreneurial activity should therefore focus on the sources of opportunities and on how these opportunities are pursued. The literature on transnational entrepreneurship stresses that migrant entrepreneurs are in a favourable position to identify market gaps and opportunities in different places and across different consumer groups in order to run a business (Drori et al., 2009; Elo et al., 2018; Zapata-Barrero & Rezaei, 2019). Therefore, understanding the role of these places and groups in detail is key to fully grasping how transnational entrepreneurs pursue business opportunities.

Previous academic models and concepts in migrant entrepreneurship have not fully succeeded in recognising the role of different groups and places. This is due to a prevailing focus on the country of residence as well as a conception of migrants as members of a single coherent ethnic/national group, seizing opportunities linked to co-national/co-ethnic markets and networks (Romero & Valdez, 2016; Sandoz et al., 2021; Solano, 2016a, 2020; Yamamura & Lassalle, 2020). Excluding other groups and places from the onset has prevented scholars from fully explaining the role played by different places and different groups in the pursuit of opportunities by transnational migrant entrepreneurs (Bagwell, 2018; Sandoz et al., 2021; Solano, 2016a).

To contribute to the field of transnational migrant entrepreneurship (henceforth “transnational entrepreneurship”), this article conceptualizes the *role of multiple places and multiple groups* (including *group modes of behaviour) in the pursuit (creation, identification and seizing) of opportunities by transnational migrant entrepreneurs.* In this article, we propose a new model combining the concept of multifocality, covering the simultaneous involvement of migrant entrepreneurs in both multiple places *and* multiple groups, with group modes of behaviour as an additional dimension influencing the opportunity structure.

In doing so, this article aims at advancing the theoretical and conceptual approach to the study of transnational entrepreneurship. Starting from previous concepts and reviewing the growing literature on transnational entrepreneurship, we present a new, fine-tuned version of the mixed embeddedness approach (Kloosterman & Rath, 2001), in order to better understand how transnational entrepreneurs identify and seize business opportunities. Over the years, this mixed embeddedness model has developed into a leading perspective in sociological and migration studies of migrant entrepreneurship (Barberis & Solano, 2018). The new version of the mixed embeddedness approach presented below is based on the idea that in finding and seizing opportunities, transnational entrepreneurs also consider multiple places and groups. This is the basis of the concept of *multifocality*, which was introduced by Solano (2016a, 2016b). We advance the original mixed embeddedness model by:

- refining the concept of multifocality to better account for the most recent findings related to the concept (see Barberis & Solano, 2018);
- linking multifocality to the mixed embeddedness approach and redefining the opportunity structure of the mixed embeddedness model accordingly;
- providing further empirical insights on the concept of multifocality, given that only a few articles provide an empirically illustration of it.

We empirically illustrate the proposed model and, more specifically, its multifocal dimensions, by focusing on the case of Moroccan transnational entrepreneurs in Amsterdam. We conclude with final reflections regarding the extent to which our empirical findings relate to the theory and their possible implications for future applications of multifocality in mixed embeddedness research on transnational entrepreneurship.

## Theoretical and conceptual background

In the following sections, we discuss the mixed embeddedness approach related to the field of transnational entrepreneurship. In doing so, we illustrate some existing literature, and we highlight the main remaining gaps in the field.

### The mixed embeddedness approach to migrant entrepreneurship

The phenomenon of migrant entrepreneurship has been analysed by many scholars, resulting in multiple concepts and theories about the inclination of migrants to choose self-employment (Rath & Schutjens, 2016). The mixed embeddedness approach is an advanced theoretical framework used by scholars in migration studies and sociology to analyse migrant entrepreneurship (Barberis & Solano, 2018; Ram et al., 2017). A recent analysis found that approximately 700 academic articles have used the mixed embeddedness approach as a theoretical framework to study the phenomenon (Barberis & Solano, 2018). The approach is considered particularly advanced because it combines the individual level of the entrepreneur (personal motivations, individual characteristics and social contacts) and the structural determinants/contextual conditions (Rath & Schutjens, 2016).

**Fig. 1:**
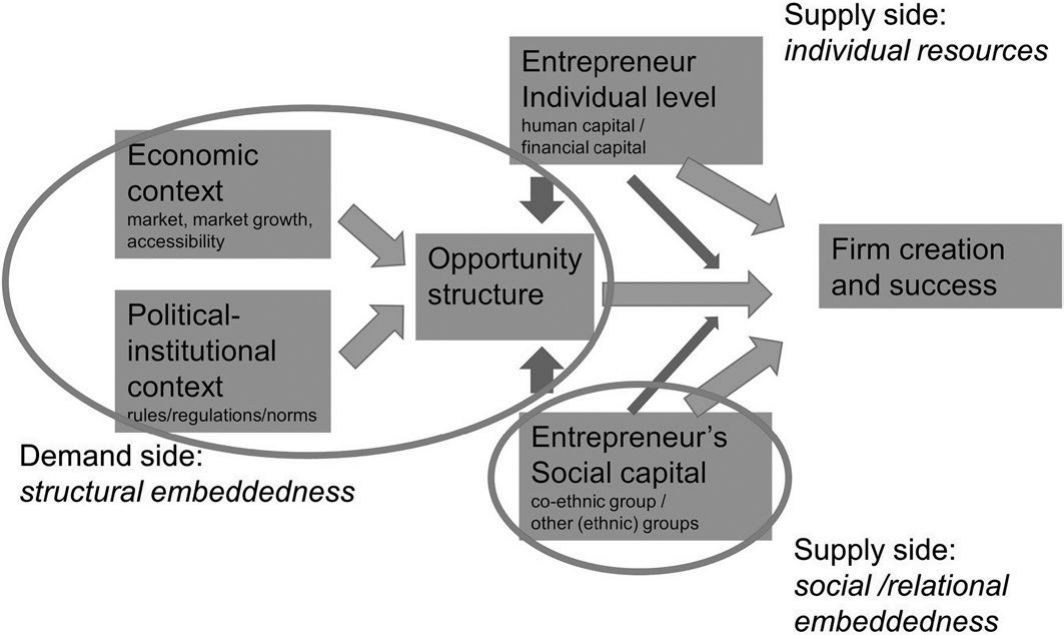
Schutjens’ mixed embeddedness model. *Source*: Schutjens (2014)

The mixed embeddedness approach was proposed by Dutch scholars Kloosterman and Rath in the late 1990s/early 2000s (Kloosterman & Rath, 2001, 2018; Kloosterman et al., 1999). Building on the Interactive model (Waldinger et al., 1985), the approach centres on the interaction between personal and group resources—mainly co-ethnic/co-national group resources—, and the contextual opportunities and conditions (opportunity structure). This interaction influences the entrepreneurial experiences and paths of migrants. Kloosterman and colleagues particularly focused on defining the opportunity structure, by emphasising that it is affected by the economic, and political-institutional contexts (as underlined by Schutjens, 2014, see Fig. 1).

The economic context refers to several conditions connected to both the overall economy and specific market conditions, such as the country’s economic phase (e.g., business cycle: growth and recession), industrial structure, market concentration, and the demand for particular products or services. The political and institutional context refers to formal acts by state and non-state entities (for instance, the central government, regional and local governments, chambers of commerce, business associations, etc.), such as sets of laws, rules, and policies that might favour or discourage entrepreneurship in general, and business transactions in particular.

The mixed embeddedness approach is particularly relevant for this article’s purpose, as it includes embeddedness in both groups and places, using the concept not only in relation to social networks, but also in terms of place-bounded institutions (Solano, 2020). The concept of embeddedness starts from the theoretical conviction that economic action is not driven solely by any individual’s rational reasoning regarding profit maximisation or economic calculations; on the contrary, it is strongly structured by social contexts such as networks, institutions, norms and values (Mingione, 2006).

Although the mixed embeddedness approach provides a relevant theoretical reference for transnational entrepreneurship, the ‘place’ aspect, which is particularly relevant for transnational entrepreneurs, has not yet been fully explored (Räuchle & Schmiz, 2019; Sandoz et al., 2021; Schmoll, 2012). Although some applications of the approach have referred to opportunities at an international level (e.g., Rath, 2002), overall, albeit with some exceptions, the approach tends to consider only the context opportunities and the entrepreneurs’ contacts which are located in the country of residence (Barberis & Solano, 2018). In doing so, the literature has generally disregarded resources and opportunities linked to countries other than the country of residence (Solano, 2020).

Furthermore, in terms of its focus on groups, the mixed embeddedness approach, and, more generally, the sociological literature on migrant entrepreneurship (e.g. the culturalist and the ethnic enclave approach, as well as the interactive model), tends to consider migrant entrepreneurs as members of a more or less coherent ethnic or national minority group (Lassalle & McElwee, 2016; Rath, 2002; Rath et al., 2020; Romero & Valdez, 2016; Yamamura & Lassalle, 2020). For example, regarding the opportunities seized by migrant entrepreneurs, the focus of previous research has been on the role and the characteristics of the so-called ethnic group (co-national group), in particular the entrepreneurial attitude or consumer behaviours of an ethnic minority group (e.g., Korean and Chinese communities). Researchers have acknowledged the relevance of co-ethnic resources and groups of co-nationals (Rath & Schutjens, 2016). However, the over-ethnised approach, which rests on the assumption of the primacy of ethnicity, has hindered the full consideration of the role of other groups, multiple identities, feelings of belonging and other dimensions such as gender and class (Brubaker, 2002; Lassalle & McElwee, 2016; Romero & Valdez, 2016). In this paper, we do not aim to enter the debate on the reification of concepts such as group or identity, which goes beyond the scope of this paper (see: Brubaker, 2002; Brubaker & Cooper, 2000). Rather, we stress the fact that it is conceptually and empirically problematic to consider ethnicity/ethnic group as the only condition that influences migrant entrepreneurs’ paths.

### Transnational migrant entrepreneurship

Some scholars in the field of transnational migrant entrepreneurship have already tried to tailor the analysis, both conceptually and empirically, to the phenomenon of transnational entrepreneurs.

Chen and Tan proposed an ‘integrative model’ (Chen & Tan, 2009), which analytically explains participation and involvement in transnational entrepreneurship. The integrative model follows the mixed embeddedness approach as it ‘takes into account factors at the macro (contextual characteristics), meso (social networks), and micro (human capital) levels (Chen & Tan, 2009: 1081). The main contribution of the integrative model is to consider the effect of these characteristics (in terms of institutional setting, market conditions and government policies) and the contacts in *both* the country of origin *and* the country of residence on participation in transnational entrepreneurship.

As such, Chen and Tan’s model adopted a dual perspective by focusing only on the country of origin-country of residence dichotomy. This follows an empirical tradition of articles on transnational entrepreneurship that focus on the interplay between the country of origin and the country of residence (Kwak & Hiebert, 2010; Miera, 2008; Morawska, 2006; Patel & Conklin, 2009; Portes et al., 2002; Sequeira et al., 2009). For example, Sequeira et al. (2009) analysed the embeddedness of transnational entrepreneurs in their countries of residence and of origin. They found that positive perceptions of the opportunities in the country of residence and greater embeddedness in the country of origin influence the types of businesses and their success. In this, they neglect the possible relevance of perceived opportunities in other (third) countries. Similarly, Patel and Conklin (2009) analysed how U.S. Latin American transnational entrepreneurs balance their networks in order to be able to operate in two institutional settings (the U.S. and their country of origin). Although their analysis is interesting with regard to understanding the effect of balancing contacts from different contexts on the identification and exploitation of business opportunities, they do not consider the possibility that these networks might have proved to be unbalanced if contacts from other countries had also been addressed.

Thus, the conceptual and empirical analyses have mostly been limited to the country of residence and the country of origin. There are exceptions to this trend of including two countries in the analysis—especially in more recent years (Bagwell, 2015, 2018; Jones et al., 2010; Solano 2016a, 2016b, 2019; Sommer & Gamper, 2018; Wong, 2004). Among these exceptions, Sommer and Gamper (2018) showed that migrants from the former Soviet Union in Germany developed different transnational business activities in both their ethnic niche and the mainstream market. These activities involved their country of origin as well as other countries. Similarly, Bagwell (2018) found that Vietnamese entrepreneurs were influenced by their mixed embeddedness in the institutional and economical settings (transnational opportunity structure) of the places where the Vietnamese diaspora was located and in their diasporic networks. In conclusion, we might say that there is increasing interest in, and empirical evidence for, including multiple places in the analysis of transnational entrepreneurship. Despite these few exceptions, as stressed by Sandoz et al. (2021), it is necessary to expand the understanding of how transnational migrant entrepreneurs develop connections and seize opportunities linked to multiple locations.

Furthermore, when it comes to the role of groups, the limitations in the field of transnational entrepreneurship mirror those in the general field of migrant entrepreneurship. The literature on transnational entrepreneurship adopts the same (limited) focus, as it tends to consider migrant entrepreneurs mainly as part of an ethnic or national minority group. Previous articles have often focused on the role of relatives and the diaspora, in terms of customers, employees and supporters (Lassalle & McElwee, 2016; Moghaddam et al., 2018). In this way, when it comes to the role of groups, it is suggested that transnational entrepreneurs mainly refer to co-nationals/co-ethnics. For example, Pruthi et al. (2018) illustrated the case of Indian transnational entrepreneurs by focusing on the role of their professional and personal ‘ethnic’ ties (Indian contacts). Bagwell (2018) focused on the role of the Vietnamese diaspora and the contacts that entrepreneurs entertain with them. As already stated in the section on migrant entrepreneurship in general, this focus undervalues the role of other groups in the entrepreneurial process. An exception to this trend is represented by De Luca and Ambrosini (2019), who took into consideration the role of mixed networks (with other foreigners and/or natives) in their analysis of the strategies of female migrant entrepreneurs in Italy.

In conclusion, despite some recent conceptual and empirical attempts to go beyond the ‘country of residence-country of origin’ dichotomy and its focus on the co-ethnic/co-national group, the field of transnational entrepreneurship remains conceptually and empirically underdeveloped when it comes to shedding light on entrepreneurial opportunities through contacts with multiple countries and multiple groups (Sandoz et al., 2021; Solano, 2020).

## A multifocal approach to mixed embeddedness

Starting from the abovementioned critical points, we now introduce our multifocal model to better assess the role of groups and places (i.e., the relationships with third countries or with people who are not co-nationals) in the pursuit of business opportunities by transnational entrepreneurs. We advance the theoretical and conceptual state of the art on transnational entrepreneurship by fine-tuning the mixed embeddedness approach. We do so by considering the concept of multifocality and through the inclusion of group modes of behaviour. In what follows, after introducing the concepts of multifocality and group modes of behaviour, we illustrate the new features that we add to the mixed embeddedness approach.

### Multifocality

In our model, we start from the concept of *multifocality*, originally introduced by Solano (2016a). Vertovec (2004), who uses the term ‘focality’ to refer to the membership and everyday practices of migrants, developed the concept of bifocality in reference to migrant transnationalism. As such, bifocality refers to a dual orientation, namely, the fact that the lives and actions of migrants are influenced by both their context of immigration (country of residence) and their context of origin (country of origin). This dual orientation influences their actions and decisions.

Solano (2016a, 2016b) expanded this by introducing the term *multifocality* and applying it to both places and groups. The author defined multifocality as ‘the structural and relational embeddedness of migrants in places and groups’ (p. 176), and referred to embeddedness as ‘the degree to which immigrants’ actions are influenced by their involvement in places and/or groups. Saying for example that ‘immigrants are embedded in their co-national group’ means that their enmeshment in their group of co-nationals effectively conditions their actions and decisions’ (p. 176).

The idea is that migrant entrepreneurs are not always linked to only two places and only one group; rather, their views, feelings of belonging and identities are or become multiple and multi-sited (Ehrkamp & Leitner, 2006; Romero & Valdez, 2016; Solano et al., 2020; Vacca et al., 2018). Although migrant entrepreneurs live in their new country of residence, in everyday business life, they refer to multiple places and groups, e.g., market demands of natives, support from family contacts living in their countries of origin, their countries of residence or elsewhere, trade regulations of the country of origin, etc.

Compared to Solano (2016a), we re-define multifocality as *simultaneously taking into account multiple places and multiple groups*. In our definition, *multifocality* means that multiple places and multiple groups are considered reference points for the migrants’ entrepreneurial actions.

We expand Solano (2016a)’s definition by including the possibility that migrant entrepreneurs can consider places and groups without being embedded in these contexts. For example, migrants can connect their business with others in their country of origin, in their country of residence, and with others in a third country. They can for instance have business relations with shop owners in their country of origin to export certain products from their country of residence, and have relations with suppliers in a third country to import other products from this country. In such cases, we can expect that they are in contact with third countries without being embedded in them. Our definition of multifocality covers this possibility. Solano (2016a) confirmed the likelihood of this situation. He found that, although Moroccan import/export entrepreneurs took into account, and had social contacts with, people in different countries (i.e., Morocco, Arab countries, and European countries), they were primarily embedded in Morocco (their country of origin) and their country of residence.

We argue that multifocality means that migrant entrepreneurs take into account, and have business connections, with multiple *places* and multiple *groups* when it comes to pursuing business opportunities.

First, entrepreneurs can refer to *different countries*—the country of origin, that of residence and other third countries. Following the recent literature on transnationalism, in including third countries we also refer to the possibility that those connections could go beyond the diaspora (Levitt & Jaworsky, 2007; Vacca et al., 2018).

Secondly, entrepreneurs can also take into account *different groups or subgroups*—and their characteristics. When we talk about groups, we do not intend to use them as a taken-for-granted concept (Brubaker, 2002; Brubaker & Cooper, 2000). Instead, we analyse how the entrepreneurs use their links and how they take into consideration the characteristics of different groups simultaneously. Our critique concerning groups departs from the point that the main focus of the literature on migrant entrepreneurship is the so-called co-ethnic group, and in our model we include the influence of multiple/additional groups based on people’s ethnic or national background. We also add the family as the literature has sometimes conflated family with ethnicity (Valdez, 2016).

Please note that we do not disregard the importance of other group segmentations such as class and gender. Advanced approaches to the topic stress the importance of employing an intersectional approach that considers how class, gender and other factors influence access to, and seizing of, opportunities (Romero & Valdez, 2016). This relevant topic is indeed included in the mixed embeddedness approach, which considers the interplay between contextual conditions and opportunities, and individual and group resources (Kloosterman, 2010; Kloosterman & Rath, 2001; Rath & Schutjens, 2016). As such, individual entrepreneurial characteristics are already part and parcel of the original model. Including other groupings goes beyond the focus of this paper. Rather than focusing on how migrant entrepreneurs seize these opportunities in general (Solano, 2020), we emphasise the role of multiple places and multiple groups in the creation (and seizing) of business opportunities (the opportunity structure).

### Multifocality and opportunity structure

Regarding both places and groups, migrant entrepreneurs may consider place features as well as group behaviours and attitudes. In what follows, given the article’s aim, we focus on how multiple places and groups contribute to create the opportunity structure, thus zooming in on the left part (the opportunity structure) of the mixed embeddedness model (see Fig. 1).

**Fig. 2:**
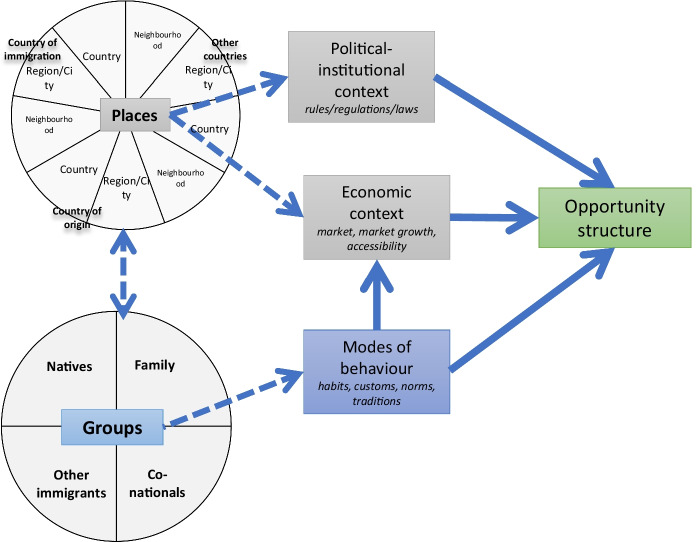
A multifocal approach to mixed embeddedness—the opportunity structure

Figure 2 illustrates that migrant entrepreneurs may run businesses that take into consideration, and are connected to, multiple different places (multifocality regarding places). The places’ characteristics and peculiarities—in terms of their political-institutional context and economic content—affect the opportunity structure. Therefore, in comparison with previous models which refer mainly to the country of residence or, at most, the country of origin, we underline that the political-institutional and economic context of multiple places influence the opportunity structure. For example, Bagwell (2018) showed how the institutional regimes, economies and markets in key countries of the Vietnamese diaspora (e.g., Czechia and Poland) influenced business development. Post-communist economic liberalisation policies in those countries generated a request for western goods and services. This led some London-based Vietnamese entrepreneurs to open branches of their nail salons in those countries.

Besides the different places, entrepreneurs are also connected to different groups (multifocality regarding groups). The connections of transnational entrepreneurs with multiple groups create opportunities. For example, Ambrosini (2012) presented the case of transnational shops offering ‘ethnic’ products. These shops target both migrants, who require products from their countries of origin, and natives, who are attracted by the exoticism, the price or the quality of these products. In this case, the connections with two groups (migrants and natives) bring to light opportunities for transnational ethnic shops.

The interplay between places and groups is a crucial basis of migrant entrepreneur multifocality. In fact, groups and places are inextricably related, as all people live in socio-spatial/geographical contexts. The combined characteristics of places and groups produce new conditions and behaviours (see for example, Salih, 2001) that influence entrepreneurship. Depending on their geographical location, groups can play a diverse role in promoting different values and customs, which in turn creates new business opportunities. For example, in his research on Moroccan entrepreneurs in Amsterdam and Milan, Solano (2016a) showed that Moroccans require different types of products, depending on whether they live in Morocco (as natives) or in another country (as migrants). In the first case (Moroccans living in Morocco), they require Italian products (e.g., foods, suits, shoes). In the second case (Moroccans abroad), they require products linked to their origin (e.g., typical Moroccan foods, Arab clothes). Such differences generate various opportunities for Moroccan migrant entrepreneurs in Italy.

Given the relevance of multiple groups, we propose to extend the opportunity structure concept by paying more attention to the ‘group’ dimension of the opportunity structure and to the interplay between this and the other dimensions that make up the opportunity structure. Previous models and approaches on migrant entrepreneurship have analysed the role of groups—or rather, of ethnic/co-national groups—in entrepreneurial opportunities (Lassalle & McElwee, 2016; Rath & Schutjens, 2016). This was generally linked to group attitudes (e.g., the entrepreneurial attitude of an ethnic minority group) and economic contexts (group characteristics and behaviours viewed as a market condition). However, in the mixed embeddedness approach (see Fig. 1), the role of the group was explicitly recognised only in connection to the entrepreneurs’ social capital, i.e., the role that their contacts play as source of financial support, information and advice, or labour. Thus, the role of groups in creating the opportunity structure in the mixed embeddedness approach was still rather limited.

We therefore propose to clearly set apart the characteristics of groups in terms of *group modes of behaviour*, which contribute to the opportunity structure (Fig. 2). We define group modes of behaviour as the *set of habits, attitudes, inclinations, and role models distinctive to a certain group*. Examples of group modes of behaviours can be found in both consumption habits and entrepreneurial conduct, such as the Italian propensity to become self-employed workers in the food sector, or the British habit of drinking tea. Those modes of behaviour create opportunities that transnational entrepreneurs can seize. For example, Kwak and Hiebert (2010) analysed the case of transnational Korean language schools in Canada. The custom of Korean people going to Canada or the U.S. to learn English provided Korean migrant entrepreneurs in Canada with a business opportunity in educational services.

Figure 2 shows that the opportunity structure is affected by the following three dimensions: (1) the economic context and (2) the political-institutional context, which are both related to different places; and finally (3) the modes of behaviour of the different groups involved in the entrepreneurial endeavour.

Group modes of behaviour affect the opportunity structure through the conditions of the market in which entrepreneurs develop their business (the economic context). An example of group modes of behaviour is the tendency to cluster in a sector (e.g., in Italy, Egyptians in take-away pizzerias). This creates unfavourable market conditions, as entrepreneurial concentration in one sector among specific groups likely increases competition.

## Transnational migrant entrepreneurs in Amsterdam: an empirical illustration of our conceptual contribution

In this section, we present the case of Moroccan transnational entrepreneurs in Amsterdam and we illustrate the extent to which different places and different groups are involved in the pursuit of business opportunities. After presenting the methodological approach and describing the sample, we explain in detail how respondents identify and seize entrepreneurial opportunities, and we highlight which places and groups are involved in these processes, and to what extent. As such, we address the relevance of multifocality and group modes of behaviour for transnational entrepreneurship.

### Background, methods and sample description

The study on which this article is based focuses on first-generation Moroccan entrepreneurs running a transnational business in Amsterdam.

Amsterdam and the Netherlands have 60 years of history as places of immigration. The Moroccan group is traditionally one of the most important groups in the Netherlands, next to people with Suriname and Turkish backgrounds (Bijwaard, 2010; Rath, 2009).^1^ Since the 1960s, many Moroccans have migrated to the Netherlands. In subsequent years, the lacklustre economic situation of Morocco further pushed them towards Europe and, in particular, the Netherlands (De Haas, 2007). Moroccan migrants and their descendants are still one of the most relevant groups in Amsterdam. According to the *Dutch Centraal Bureau voor de Statistiek* (CBS), 77,210 people of Moroccan descent lived in the Amsterdam area in 2020 (8.8% of the total population). In Amsterdam, foreign entrepreneurs are about 33% (Rath & Eurofound, 2011), but unfortunately, data sorted by nationality are not available for the city. However, as an indication, there are 8400 Moroccan entrepreneurs in the Netherlands (i.e. 0.6% of all entrepreneurs), according to CBS figures.

The study used a qualitative approach, employing face-to-face interviews. A purposive sample was chosen based on qualitative typologies (Silverman, 2013), which means that entrepreneurs were selected based on different types of business within the category of Moroccan transnational entrepreneurs.

In order to render a more complete picture of Moroccan entrepreneurial activities, we used different methods and sources to identify respondents: (1) a non-exhaustive database list of businesses provided by the Chamber of Commerce indicating the type of business activities (but not sorted by nationality),^2^ (2) contacts from Moroccan associations with a relevant role in the Moroccan group (e.g., Moroccan business networks and Islamic cultural associations), (3) entrepreneur business cards left in shops as advertisements, and (4) the visibility of the business at street level. On some occasions, when walking around city areas with a high density of Moroccan businesses, we noticed a particularly interesting business for the research purpose (e.g. import/export) and we simply asked the owner for an interview.

By means of these strategies, 15 first-generation Moroccan entrepreneurs running a transnational business in Amsterdam were interviewed. As underlined by Harima and Baron (2020), there is a certain lack of clarity when it comes to defining the concept of ‘migrant entrepreneur with a transnational business’. To identify transnational entrepreneurs, we combined the approach of Rusinovic (2008) and that of Portes et al. (2002). In particular, following Rusinovic’s remarks, we opted for a general approach without stressing the fact that transnational entrepreneurs have to travel abroad at least twice a year, as in Portes et al. (2002). However, I used a key question asked by Portes et al. (2002), i.e. ‘Is there a relevant part of your business related with your country of origin or with other countries outside Italy/Netherlands?’ In order to avoid bias linked to self-reporting, the answer to this key question was discussed in depth with the interviewees so as to ensure the validity of the response. It is important to underline the fact that, in essence, this question “filtered” migrant entrepreneurs with relevant connections abroad. We intentionally did not limit “connections abroad” to the country of origin (Morocco in this case) or other countries, in order to potentially include a wide range of cases (bi- and multi-focal transnational migrant entrepreneurs).

This number of interviews proved sufficient to understand the entrepreneurial patterns and mechanisms of the target group, as the interviews disclosed some recurrent patterns and mechanisms. The interviews were conducted from March to November 2014, and lasted from one hour and a half to three hours.

Besides socio-demographic information, questions regarding the entrepreneurial experience, daily working practices, business links, and resources used were investigated. In line with the theoretical framework explained above, our research design included questions on the role of conditions and contacts located outside the country of residence and the country of origin, as well as outside the “ethnic” community. The research design also allowed us to identify from exactly what groups or places opportunities were identified and seized (and how).

Although the data are not very recent, we believe that they still have the potential to provide insights into a phenomenon that is now even more relevant due to further technological developments, e.g. social media and new technologies which are critical for (transnational migrant) entrepreneurs (Andreotti & Solano, 2019).

The data collected in 2014 stems from extensive interviews, which often involved multiple visits with the respondents. The interviews aimed at identifying and unravelling transnational links. The data collection and interview guide were designed to test the conceptual proposals introduced in this paper. Therefore, these data are unique in providing an empirical illustration of this article’ conceptual contribution. They reveal clear patterns and mechanisms linked to the proposed concepts.

In keeping with population trends, which emerged from the preparatory work both before the fieldwork and during the fieldwork (see Solano, 2016b for additional information), we interviewed people from two main types of transnational businesses: import/export businesses and consultancy agencies (for mediation and counselling). Out of 15 respondents, we interviewed eight from import and/or export businesses and seven from consultancy agencies. Respondents were mostly men (12/15). They had a medium–high level of education (13/15). Compared to other European countries (e.g., Southern European countries where the Moroccan migrant group is present), the peculiarity of the sample is indeed the rather high-level of education and the fact that many respondents attended secondary school and/or university in the Netherlands. Many interviewees studied at a Dutch *Hogeschool* (University of Applied Sciences), allowing them to better navigate the Dutch context. However, this is consistent with the most recent OECD figures (2017) on migrant entrepreneurs in the Netherlands.

### Findings

In what follows, we limit the scope of the empirical findings to multifocality and modes of behaviour. The contribution of the empirical part of this paper is to combine the concept of multifocality, covering the simultaneous involvement in multiple places and groups, with group modes of behaviour as one of the dimensions influencing opportunity structure, and in particular market opportunities, rather than empirically test the mixed embeddedness approach for transnational entrepreneurs (on this, see: Bagwell, 2018; Solano, 2020).

### Multifocality regarding different places

The Moroccan entrepreneurs in our sample are involved in business activities with both their country of origin (Morocco) and one or many other countries (see Table 1). In order to create and seize opportunities, respondents bridge different countries at the same time: The Netherlands and at least two other countries, often Morocco and another country. A relevant number of the respondents (7/15) have contacts with three or more other countries, including Morocco in six of the seven cases (one interviewee has no business links with Morocco). The importance of Morocco is generally high, as 13 out of 15 respondents have business contacts with Morocco. Apart from Morocco, respondents have business contacts with the Arab Peninsula (United Arab Emirates and Saudi Arabia), and European countries with a large number of Moroccan migrants (Belgium, Germany, Italy and Spain—surprisingly France was mentioned less frequently).

**Table 1:** Countries respondents have business links with (next to/apart from their current country of residence, i.e. the Netherlands)

Links	N
Only with Morocco	3
With both Morocco and one other country	4
With both Morocco and two other countries	1
With both Morocco and three or more other countries	5
Only with other countries (excluding Morocco)	2
**Total**	**15**

The majority of respondents are multi-focal when it comes to places, meaning that they take into consideration more than two places for their business. The choice of countries is mainly linked to the market opportunities that they become aware of, as also suggested by other studies (Rusinovic, 2008; Solano et al., 2020; Vershinina et al., 2019).

First, import/export businesses connect the production of specific products in one or more countries, with a demand for these products in other countries. In this case, the links are often based on similarity, according to the respondents. The main countries that respondents have contacts with have a population—or, at least, part of their population—that have habits and traditions (modes of behaviours, see below) similar to the Moroccan ones. In some countries, similar goods are produced or consumed, while in others, many North African migrants are present, and this represents a perfect reference for import/export.

To identify and seize market opportunities in those countries, respondents do not only rely on their family or friendship networks or their already existing contacts, they also actively search for new key contacts. J. (A20), who provides fabrics and curtains for interior decorating, is a case of the former. He found “information and contacts for the business from friends in Morocco”. By contrast, R. (A13), who imports foods from abroad for a clientele of other migrants, relied more on his own networking skills:

When I started, I knew that here there are many migrants and an ethnic marked, so I decided to exploit these possibilities by importing products from abroad and selling these products to other shops. But I started without any contacts and I built them by visiting a lot of international food fairs. Now I have a lot of contacts around the world, but I started from zero.

Second, consultancy businesses often match the economic situation of one country with the will of entrepreneurs in another country to internationalise their business. In this case, the link with their family networks and with the diaspora is even weaker. Respondents conducting a transnational consultancy business rely more on direct knowledge of the contexts where they operate—often in connection to previous life and work experience. For example, S. (A04), who organises study trips abroad for Dutch students, chose Morocco and Jordan because “*I had reliable connections over there. I lived there for a long time, so I built up a huge network over there; it was easy, because I lived there*”.

B. (A14) has a consultancy agency that helps companies from MENA (Middle Eastern and North African) countries, in particular from the Arab Peninsula, to buy vehicles (trucks and vans) from the Netherlands and Germany, two countries where the production of these goods is particular advanced. He “*worked for six years as an account manager in two trucking companies, both times in a department dealing with the Middle East and North Africa*”. Thanks to this and to his language skills, he “*acquired the knowledge and the contacts that I needed for the business*”. Another example is A. (A11). Since Morocco is his country of origin and he knows “*how to go about searching for information there*”, he started a business supporting companies that wish to enter the Moroccan market.

Considering multiple different places is fundamental for respondents to create, identify and exploit market opportunities. As also shown by Villares-Varela et al. (2018), respondents follow a typical process known in entrepreneurship theory as bricolage, namely ‘making do by applying combinations of the resources at hand to new problems and opportunities’ (Baker & Nelson, 2005: 333). Respondents link different countries to combine resources and create opportunities.

S. (A26), who owns a decorating company, and A. (A05), who retails Moroccan dresses and perfumes to a clientele of co-nationals, are two cases in point:

My company provides flower ornaments and decorations for luxury hotels and restaurants. I decorate one hotel and a number of restaurants here in Amsterdam; some of my other clients are in Casablanca (hotels) and Antwerp (restaurants). In Belgium, the luxury business is more developed than in the Netherlands. I also provide flowers to some hotels in Dubai. The flowers are from the Netherlands of course, and the decorations (like the vases for the flowers) are made in Italy and Spain. Another product I import is Argan Oil from Morocco, which I sell in the Netherlands and Dubai. You see the triple connection: the Netherlands, Morocco and Dubai: Morocco for the Argan Oil, the Netherlands for the flowers, and Dubai because people there love luxury goods and excellent products. This triad is the key to my business and my success. Belgium is also important to me, but these three places are fundamental. (A26)

The products I sell are from my country of origin and Saudi Arabia. Most of the products I sell are only obtainable there, some in Morocco and some only in Saudi Arabia. It would be much more difficult to get these products here in the Netherlands. (A05)

These two examples clearly illustrate the key role of multifocality for import/export businesses. A. (A26) would not have been able to seize opportunities in the Netherlands and Morocco without his business contacts in other countries. Similarly, A. (A05) imports most of the goods from Morocco and Saudi Arabia because these products are not available in the Netherlands.

In the case of consultancy businesses, besides Morocco, they target other MENA (Middle East North Africa) countries since they speak Arabic. They connect more than two countries in order to seize identified opportunities to conduct their business. For example, A. (A09) owns an “*Amsterdam-based global Communication, Marketing and Design company with a satellite office in Casablanca (Morocco), operating in Europe as well as in the MENA region*”. A. chose to have an office in Morocco to facilitate business links with North-African countries, but he provides his services to a clientele that goes well beyond Morocco.

To sum up, the interviewees’ stories clearly highlight the fact that two countries are often insufficient for the purpose of conducting their business. Therefore, multifocality in different places (and not only bifocality) plays a relevant role in the pursuit of market-related business opportunities. Regarding the mechanisms that enable multifocality, respondents were able to identify and seize opportunities in several places thanks to their personal initiative, contacts and research, as well as their human capital in the form of previous work experience and language skills (on this see also: Solano, 2020).

### Multifocality regarding different groups

The characteristics of groups, together with those of places, represent an opportunity for respondents to exploit. However, in most of our cases (9/15), respondents do not target a particular group. This means that, in most of the cases, they do not consider the characteristics and modes of behaviours of a specific group and, therefore, they do not seize opportunities that are linked to a specific group.

Furthermore, respondents do not generally combine resources and opportunities linked to different groups of people (e.g., natives and co-nationals) and, therefore, they do not seems to be multifocal when it comes to groups (Table 2). Only in one case does the respondent target more than one group, by focusing on both co-nationals and other migrants. R. (A13) imports low-cost products (oil, biscuits and fresh foods) for migrants in Amsterdam:

Here in the Netherlands there is a big ethnic community and I bring from abroad some low-cost products for my co-nationals and other migrants.

**Table 2:** Target customers

Target customers	N
Co-nationals or Arab migrants	5
Only other groups (no co-nationals or Arab migrants)	0
Co-nationals (or Arab migrants) and other groups	1
No specific target group	9
**Total**	**15**

When they focus on one or more groups (6/15), respondents mainly refer to opportunities connected to features of their co-nationals and, as a consequence, of the Arabic-speaking groups (5/15). They mainly focus on the Moroccan group, but they also attract other Arab speaking migrants. According to the respondents, this is linked to the fact that migrants from North Africa or Arab and/or Islamic countries often have similar needs (e.g. buying *halal* meat). However, their initial business idea was to capitalise on a need of the Moroccan group, and their focus remains on this group. This is clearly explained by A. (A18), who sells traditional female clothing. As there is a large Moroccan community in Amsterdam, she decided to start a business in this sector:

This business is mainly for Moroccans. They are my main customers. They are the people who are interested in these products. When I started I thought of my co-nationals, because I decided to offer Moroccan clothes. But there are many people from other North-African and Arab countries who also come to my shop. (A18)

N. (A08) is another case in point. He sells Arab dresses and traditional clothes to a clientele of co-nationals, but also to people from other North-African countries. They have a need for these clothes and N. satisfies their demand: “*here in Amsterdam and in the Netherlands, there are many Moroccans and North-African migrants. (They) want to dress like they do in Morocco in some special events, so we provide them with the things they need*”.

### Group modes of behaviours

As the examples illustrated in the previous section show, the relevance of co-nationals for creating business opportunities is linked mainly to modes of behaviour. Group modes of behaviour contribute to creating an opportunity structure that respondents take advantage of.

Market opportunities emerge in connection with the customs, or modes of behaviour, of co-nationals. In this regard, the concentration of a certain group in a certain place usually provides the entrepreneurs with a market where they can sell the desired products. This holds especially true for import/export businesses. The modes of behaviour (needs, customs, etc.) of co-nationals (and other migrants) who are concentrated in certain places (a neighbourhood, for example) create markets that are usually easy for Moroccan import/export entrepreneurs to tap, as underlined in the literature (Hiebert et al., 2015; Kloosterman et al., 1999; Wilson & Portes, 1980). This, for example, is stressed by A., who owns a shop selling Moroccan dresses and perfumes: “*Amsterdam is a large city, where a lot of Moroccans live. That’s my market!*” (A05). S. (A24) is the only case of a consultancy agency that seizes the opportunity created by his co-nationals’ modes of behaviour. He runs a website with all kinds of information (housing, leisure and travels, business) about Morocco. The website targets second- or third-generation Moroccan-Dutch people:

They grew up in a North European culture but they spend a lot of time in Morocco, to visit friends, relatives etc. They usually also have some problems with the bureaucracy and the law when they want to buy a house or if they have to bury a relative who dies in Morocco. And they have no knowledge of the rules or the way to do this. (A24)

Their focus on the Moroccan group leads respondents to internationalise their business and to adopt a multifocal approach concerning places. This happens in many ways, such as exporting or importing products—as in the case of import and/or export businesses (e.g., the previous examples of A05, A08, A18), or providing information that the group needs—as in the case of consultancy agencies. For example, J. (A20) provides fabrics and curtains for interior decorating. His co-nationals require these specific products when they wish to create a “*Moroccan atmosphere*”, and Moroccan people in Amsterdam want *“a little bit of Morocco in their home”*. He imports from Morocco and Turkey because the fabrics are different from what he would be able to find in the Netherlands. The above-mentioned case of N. (A08), who sells Arab dresses and traditional clothes to a clientele of co-nationals/North-African migrants, shows how the link with this group can lead to a multifocal approach concerning places. First, he maintains links with Morocco since he needs to import materials and formal Arab-style clothes from Morocco. Second, he has Moroccan and North-African customers from other countries where the Moroccan/North-African diaspora is located (i.e., Belgium).

In conclusion, our findings show that multifocality regarding places contributes to the creation, identification, and exploitation of key business opportunities. By contrast, having contacts with multiple groups (multifocality regarding groups) does not seem to play a relevant role in the pursuit of business opportunities. However, we found that group modes of behaviour create business opportunities for Moroccan transnational entrepreneurs in Amsterdam. Furthermore, as the Moroccan diaspora is spread all over Europe, seizing opportunities created by co-national modes of behaviours has led entrepreneurs to be multifocal regarding places.

## Discussion and conclusion

To better understand the creation, identification, and seizing of available opportunities by transnational entrepreneurs, in this article we use the notion of multifocality and link it with the definition of opportunity structure in the mixed embeddedness approach.

We revise Solano (2016a, 2016b)’s definition of multifocality to better account for the most recent findings related to the concept and the topic (see Barberis & Solano, 2018). We define multifocality as *simultaneously taking into account multiple places and multiple groups*. The views and identities of migrant entrepreneurs are or become multiple and multi-sited. Therefore, *multifocality* means that migrant entrepreneurs refer to multiple places and multiple groups for their entrepreneurial actions. This definition calls for renewed attention to multiple places and groups, to go beyond the common focus on the ‘country of residence-country of origin’ dichotomy and excessive attention to the co-ethnic/co-national group. By doing this, we advance the field of transnational entrepreneurship, which remains conceptually and empirically underdeveloped, to shed light on entrepreneurial opportunities created by other places (e.g., third countries) or groups.

The concept of multifocality, in connection with that of opportunity structure, allows us to refine the mixed embeddedness approach for the study of migrant entrepreneurship in a globalised world. When it comes to the political-institutional and economic context, we not only stress the conditions of the country of destination, but also of other places that entrepreneurs are linked to. In addition, to further highlight the role of groups, we expand the opportunity structure by introducing the concept of *group modes of behaviour* as a third area contributing to the opportunity structure. We define group modes of behaviour as the *set of habits, attitudes, inclinations, and role models distinctive to a certain group*. Thus, the opportunity structure is now influenced by not only the political-institutional context (laws, regulations, rules) and the economic context (e.g., market conditions)—the two spheres that are already covered by the mixed embeddedness approach—, but also by a third sphere: that of group modes of behaviour (habits, customs, norms, etc.).

The concept of multifocality also opens up new methodological avenues in academic research. This entails considering not only the contexts of residence and of origin, but also the possibilities that entrepreneurs are “players” in more than two countries and that this goes beyond the diaspora. In this respect, we stress that future empirical research designs should acknowledge the migrant entrepreneurs’ contacts located outside their country of residence and their country of origin, and their respective value for the business, and should identify exactly where opportunities are identified and seized. This implies that more detailed interview designs should be developed, enabling accurate measurement of the role of multiple groups, places and contexts in transnational entrepreneurship, as well as their actual value for specific parts of the entrepreneurial venture.

We also provide an empirical illustration of the conceptual exercise. The findings from Moroccan transnational entrepreneurs in Amsterdam (partially) confirm the explanatory power of our concepts by providing insights into how multifocality can shape entrepreneurial opportunities. By looking outside their country of residence, transnational entrepreneurs combine features from several places, creating and seizing market opportunities for their business. Rather than considering only their country of residence (in our case, the Netherlands) and their country of origin (Morocco), they also focus on third countries, so they usually combine more than two countries. The extension to other countries is sometimes, but not always, linked to the Moroccan/North-African diaspora and its modes of behaviour. Respondents are, therefore, multi-focal regarding place.

Multifocality is fundamental to link previously unconnected opportunities, which would not have been possible to seize otherwise. This might of course be linked to the specific group of respondents. Indeed, the Moroccan group presents at least two characteristics that favour their multifocality. First, they master Arabic—although some of them speak Berber as their native language—, which allows them to have business contacts with many different countries (e.g., MENA countries). Second, the Moroccan diaspora is scattered all around Europe and this represents a resource for the respondents, favouring the internationalisation of their business.

However, in the case of Moroccan transnational entrepreneurs in Amsterdam, multifocality actually seems to refer to *multilocality* (Koenig, 2005), as it applies only to multifocality regarding places and not regarding groups. Respondents generally consider multiple places, but merely one group, which is the Moroccan group. They mainly focus on the Moroccan group, but they also attract other Arabic-speaking migrants, given some similarities in their modes of behaviours. This result might be linked to the fact that Moroccans in the Netherlands are a particularly numerous group, and they have an extensive diaspora in Europe (De Haas, 2007). This might have led them not to seek opportunities outside their group of co-nationals.

Despite the focus on one group only, the findings confirm the importance of group modes of behaviour in influencing the economic/market dimension of the opportunity structure. Respondents take advantage of their co-nationals’ modes of behaviour, which in turn contribute to creating an opportunity structure that Moroccan transnational entrepreneurs exploit. This is key to business internationalisation, as the Moroccan diaspora is scattered throughout Europe. Furthermore, our analysis suggests that there are some differences between businesses that provide customers with services (e.g. consultancy businesses) or physical products (e.g. import/export businesses). First, multifocality in places is linked to the diaspora for import/export businesses, whereas it is linked to their language skills (knowledge of Arabic) for consultancy businesses. Second, consultancy businesses are less likely to focus on the needs and customs of a specific group of people, compared to transnational businesses that focus on physical products (import/export businesses). This seems due to the nature of the businesses: import/export businesses seize opportunities linked to the needs of people (e.g. consumption behaviours), while consultancy businesses mainly work for other entrepreneurs.

As illustrated by our empirical data, the concept of multifocality seems a promising academic framework for understanding the opportunities that are taken by transnational entrepreneurs and the process through which they do so, since it takes different groups and places into account. Table 3 provides a summary of our conceptual contribution and its methodological implications, related to the empirical findings.

**Table 3:** Conceptual and methodological implications of the theoretical model proposed and empirical findings

Conceptual contributions	Methodological implications	Empirical findings
***Multifocality***: the act of simultaneously taking into account multiple places and multiple groups		
*Multifocality in places*		
– To consider the conditions of other countries (country of origin and third countries), other than the country of destination	– To investigate the role of conditions of different contexts and how these influence the respondents’ business pattern – To ask what countries and contexts the entrepreneurs are linked to, the conditions of these countries, and how these have influenced the respondents’ business pattern	– Respondents combine features from several places in order to create or seize business opportunities for their business, the country of residence and that of origin (Morocco), but also third countries – Multifocality in places is key to linking previously unconnected opportunities
*Multifocality in groups*		
– To consider the possibility that entrepreneurs feel they belong to and/or are influenced by different groups (multi-faceted identity)	– To investigate the role of multiple groups (not only the “ethnic” group) and how these influence the respondents’ business pattern – To gather information on the role of persons belonging to different groups and the characteristics of these groups (not only the “ethnic” group)	– Respondents generally do not combine resources and opportunities linked to different groups – When they target a specific group, this is the Moroccan group – Multifocality in groups plays a minor role, compared to multifocality in places
***Group modes of behaviours***: set of habits, attitudes, inclinations, and role models distinctive to a certain group		
– To consider that specific habits and attitudes that are distinctive to a certain group might influence the entrepreneurs’ business opportunities	– To investigate the role of these habits and attitudes in reference to multiple groups – To gather information on the entrepreneurs’ perceptions of these group habits and attitudes, and how they influence their business pattern	– Respondents take advantage of these modes of behaviour (mostly from the co-national group), which contribute to create the opportunity structure that they exploit – Group modes of behaviour are key in influencing the economic/market dimension of the opportunity structure

More empirical research is needed to corroborate our proposed conceptual additions, although some empirical studies already do sustain our argument (e.g., Bagwell, 2015, 2018; Jones et al., 2010; Solano, 2016a; Sommer & Gamper, 2018). The present article has its main limitation in the fact that the findings are based on a single research population (Moroccan migrants) in one city (Amsterdam). Taking into account more national groups or different countries may lead to different results and to a more general understanding of the model (e.g., concerning multifocality in groups). Future research should explore the link between national origin and degree of multifocality, as we found that our respondents employed their Arabic language skills to reach countries other than Morocco (e.g., MENA countries). Therefore, it would be interesting to ascertain whether multifocality also applies to other national group of migrants who speak a less “international” native language. The link between multifocality in groups and in places should also be tested for other national groups with a less extensive diaspora. For example, less sizeable diaspora groups of migrants may focus on the country of origin only and on multiple groups, e.g., as potential customers. Furthermore, the data on which this article is based were collected more than 5 years ago. We believe the data are still meaningful to illustrate our concepts. The interviews providing the empirical material for this article were collected for the sole purpose of describing and understanding every transnational link of the entrepreneurs interviewed, and, in this sense, they are key to empirically sustain our conceptual contribution. In addition, the phenomenon of transnational migrant entrepreneurship is even more relevant now due to further technological developments (Andreotti & Solano, 2019). However, future research should test the proposed model amid the COVID-19 crisis. The COVID-19 crisis may have temporarily or permanently changed the involvement of migrants and migrant entrepreneurs in multiple places or groups, and affected their subsequent transnational business opportunities.

Finally, one aim of this article is to question how the literature on migrant entrepreneurship tends to reduce migrants to their belonging to one ethnic/national group. Our paper still offers a relatively homogeneous view of the studied population and does not analyse the role of other factors such as gender and class, or education or age in any depth. In subsequent studies, it would be interesting to analyse migrant multifocality with an intersectional approach (Romero & Valdez, 2016). By doing this, it would be possible to consider for example how people identifying as members of the same group occupy different social positions, and thus have different opportunities to access support and resources within this group (and beyond).

Nevertheless, this article contributes to the existing knowledge on transnational migrant entrepreneurship by proposing new concepts to refine the mixed embeddedness approach and better understand existing opportunities for migrant entrepreneurship in a globalised world. The empirical findings sustain the theoretical proposals by showing the relevance of multifocality and group modes of behaviour for the seizing of business opportunities by migrant entrepreneurs.

## Data Availability

Not applicable.

## Footnotes

1 https://opendata.cbs.nl/statline/#/CBS/nl/dataset/37296ned/table?ts=1604499051197.

2 We looked for businesses owned by Moroccan immigrants by using (as keywords) the names of major Moroccan cities (e.g., Casablanca, Rabat) and the words ‘Morocco’ ‘Moroccan’ ‘Moroccans’, and combining them with other keywords such as ‘import’, ‘export’, ‘international’ (of course, in the Dutch translation).
